# Investigating the Origin of Mycobacterium chimaera Contamination in Heater-Cooler Units: Integrated Analysis with Fourier Transform Infrared Spectroscopy and Whole-Genome Sequencing

**DOI:** 10.1128/spectrum.02893-22

**Published:** 2022-10-12

**Authors:** F. Bisognin, F. Messina, O. Butera, C. Nisii, A. Mazzarelli, S. Cristino, M. R. Pascale, G. Lombardi, A. Cannas, P. Dal Monte

**Affiliations:** a Microbiology Unit, IRCCS Azienda Ospedaliero-Universitaria di Bologna, Bologna, Italy; b Department of Experimental, Diagnostic and Specialty Medicine, Alma Mater Studiorum-University of Bologna, Bologna, Italy; c UOC Microbiology and Bio-repository, National Institute for Infectious Diseases “Lazzaro Spallanzani”-IRCCS, Rome, Italy; d UOS Technical Health Professions, National Institute for Infectious Diseases “Lazzaro Spallanzani”-IRCCS, Rome, Italy; e Department of Biological, Geological, and Environmental Sciences, Alma Mater Studiorum-University of Bologna, Bologna, Italy; University Paris-Saclay, AP-HP hospital Antoine Béclere, Service de Microbiologie, Institute for Integrative Biology of the Cell (I2BC), CEA, CNRS

**Keywords:** absolute filter, heater-cooler units, IR-Biotyper, *Mycobacterium chimaera*, nontuberculous Mycobacteria, whole-genome sequencing

## Abstract

Mycobacterium chimaera is ubiquitously spread in the environment, including factory and hospital water systems. Invasive cases of M. chimaera infection have been associated with aerosols produced by the use of heater-cooler units (HCU) during cardiac surgery. The aim of this study was to evaluate for the first time the performance of IR-Biotyper system on a large number of *M. chimaera* isolates collected from longitudinal environmental HCUs samples and water sources from hospitals located in three Italian provinces. In addition, IR-Biotyper results were compared with whole-genome sequencing (WGS) analysis, the reference method for molecular epidemiology, to investigate the origin of *M. chimaera* contamination of HCUs. From November 2018 to May 2021, 417 water samples from 52 HCUs (Stockert 3T, *n* = 41 and HCU40, *n* = 11) and 23 hospital taps (used to fill the HCU tanks) were concentrated, decontaminated, and cultured for *M. chimaera*. Positive cultures (*n* = 53) were purified by agar plate subcultures and analyzed by IR-Biotyper platform and Ion Torrent sequencing system. IR-Biotyper spectra results were analyzed using a statistical approach of dimensionality reduction by linear discriminant analysis (LDA), generating three separate clusters of *M. chimaera*, ascribable to each hospital. Furthermore, the only *M. chimaera-*positive sample from tap water clustered with the isolates from the HCUs of the same hospital, confirming that the plumbing system could represent the source of HCU contamination and, potentially, of patient infection. According to the genome-based phylogenies and following the classification proposed by van Ingen and collaborators in 2017, three distinct *M. chimaera* groups appear to have contaminated the HCU water systems: subgroups 1.1, 2.1, and branch 2. Most of the strains isolated from HCUs at the same hospital share a highly similar genetic profile. The nonrandom distribution obtained with WGS and IR-Biotyper leads to the hypothesis that *M. chimaera* subtypes circulating in the local plumbing colonize HCUs through the absolute filter, in addition with the current hypothesis that contamination occurs at the HCU production site. This opens the possibility that other medical equipment, such as endoscope reprocessing device or hemodialysis systems, could be contaminated by *M. chimaera*.

**IMPORTANCE** Our manuscript focuses on interventions to reduce waterborne disease transmission, improve sanitation, and control infection. Sanitary water can be contaminated by nontuberculous Mycobacteria, including *M. chimaera*, a causative agent of invasive infections in immunocompromised patients. We found highly similar genetic and phenotypic profiles of *M. chimaera* isolated from heater-cooler units (HCU) used during surgery to thermo-regulate patients' body temperature, and from the same hospital tap water. These results lead to the hypothesis that *M. chimaera* subtypes circulating in the local plumbing colonize HCUs through the absolute filter, adding to the current hypothesis that contamination occurs at the HCU production site. In addition, this opens the possibility that other medical equipment using sanitized water, such as endoscope reprocessing devices or hemodialysis systems, could be contaminated by nontuberculous Mycobacteria, suggesting the need for environmental surveillance and associated control measures.

## INTRODUCTION

Mycobacterium chimaera (*M. chimaera*), identified and included in the Mycobacterium avium complex by Tortoli et al. in 2004 (1), is a slow-growing nontuberculous Mycobacterium (NTM) ubiquitously spread in the environment, including factory and hospital water distribution systems (2–4).

Waterborne pathogens may cause infections in health care facilities. Gram-negative bacteria and NTM are the most common causes of infection, although fungi, parasites, and viruses have also been implicated in nosocomial infection (5). These infections, including bacteremia and invasive and disseminated diseases, are particularly important in immunocompromised hosts and critically ill adults as well as neonates.

Transmission occurs via contact, ingestion, aspiration, or aerosolization of drinking water, or via the hands of health care workers (6). In 2013, *M. chimaera* was identified as the causative agent of invasive infections in a small number of patients undergoing cardiothoracic surgery with extracorporeal circulation. A global outbreak of infections associated with open-chest heart surgery is ongoing, and to date more than 140 cases of severe *M. chimaera* infection have been identified worldwide (7, 8).

In 2015, it emerged that the heater-cooler units (HCU) used during surgery to thermo-regulate patients’ body temperature, were contaminated with *M. chimaera* and were probably the source of infection (9). Although the water in HCU circuits does not come into direct contact with the patient or the patient’s blood, it was demonstrated that bio-aerosols were released from HCUs during surgery, leading to the airborne transmission of *M. chimaera* to patients through ventilation fans (10, 11). To prevent potential *M. chimaera* aerosolization, the HCU water reservoirs must be disinfected frequently according to manufacturer’s instructions. Chemical disinfectants are based on chlorine, peracetic acid, and hydrogen peroxide, depending on the HCU model (12, 13). *M. chimaera* is the most abundant species of NTM detected in contaminated HCUs. This could be due to its ability to rapidly form biofilm particularly on stainless steel surfaces, as shown by Siddam et al. using scanning electron microscopy (14). Furthermore, the presence of NTM biofilms in HCUs makes their sanitization extremely challenging and hospitals are still reporting intermittent *M. chimaera* positive cases of HCU water samples despite complying with the recommended disinfection procedure(s) (15, 16).

To understand the origin of HCU colonization, van Ingen and coworkers performed a phylogenetic analysis based on whole-genome sequencing (WGS) on 250 *M. chimaera* isolates from HCUs, patients and HCU manufacturing site water systems. This analysis revealed two major *M. chimaera* groups and cardiac surgery-related patient isolates were mainly classified into subgroup 1.1. Furthermore, WGS analysis showed a high level of genetic similarity between the HCU factory and surgery-related patient isolates, making a single point source contamination of the HCU during production the most plausible hypothesis (17).

Fourier transform infrared spectroscopy (FTIRS) has been proven to be an innovative and reliable approach for microbial typing. This technique analyses the biochemical composition of the microbial cells by measuring absorption of infrared light by the different (bio)molecules present in the cells. The IR Biotyper Platform (Bruker, Germany) is a FTIRS-based system suitable for microbial typing in routine practice. Since its introduction, several studies have investigated the use of IR Biotyper for typing different bacteria, especially Enterobacteriaceae and nonfermenting Gram-negative bacilli, Streptococcus pneumoniae, Staphylococcus aureus, and Legionella pneumophila (18–22). However, to date, no studies have been performed for typing Mycobacteria.

The aim of this study was to investigate the origin of *M. chimaera* contamination in HCU using integrated analysis with IR-Biotyper system and WGS analysis, the reference method for outbreak investigation. The study was carried out on a large number of *M. chimaera* isolates collected from longitudinal environmental HCUs samples and water sources in three different hospitals to investigate the origin of *M. chimaera* contamination of HCUs.

## RESULTS

### *M. chimaera-*positive samples.

Twenty-six (50%) of the 52 HCUs sampled in this study were contaminated by *M. chimaera*: 2/11 (18.2%) were HCU40 model and 24/41 (58.5%) were Stockert 3T.

Out of the 440 water samples collected during the study period, 53 (12%) tested positive for *M. chimaera* (two isolated from HCU40, 50 from Stockert 3T, and one from hospital M prefilter tap water) while two (0.5%) were positive for Mycobacterium gordonae (isolated from prefilter tap water of hospitals A and S), two (0.5%) for Mycobacterium chelonae (isolated from two different Stockert 3T of hospital A), and one (0.2%) for Mycobacterium xenopy (isolated from a Stockert 3T of hospital M).

Twenty-one of the 53 *M. chimaera-*positive water samples came from hospital A (isolated from two HCU40 and six Stockert 3T), 27 from hospital M (isolated from 16 Stockert 3T and one prefilter tap water) and five from hospital s (isolated from two Stockert 3T) (Table 1).

**TABLE 1:** Distribution of water samples included in the study

Hospital	Water samples				*M. chimaera*-positive samples			
	Total (*n* = 440)	HCU40 (*n* = 118)	Stockert 3T (*n* = 299)	Tap water (*n* = 23)	Total (*n* = 53)	HCU40 (*n* = 2)	Stockert 3T (*n* = 50)	Tap water (*n* = 1)
A	257	118	131	8	21	2	19	0
M	115	0	112	3	27	0	26	1
S	68	0	56	12	5	0	5	0

### *M. chimaera* IR biotyper linear discriminant analysis grouped for hospital origin and colony morphology.

**FIG 1:**
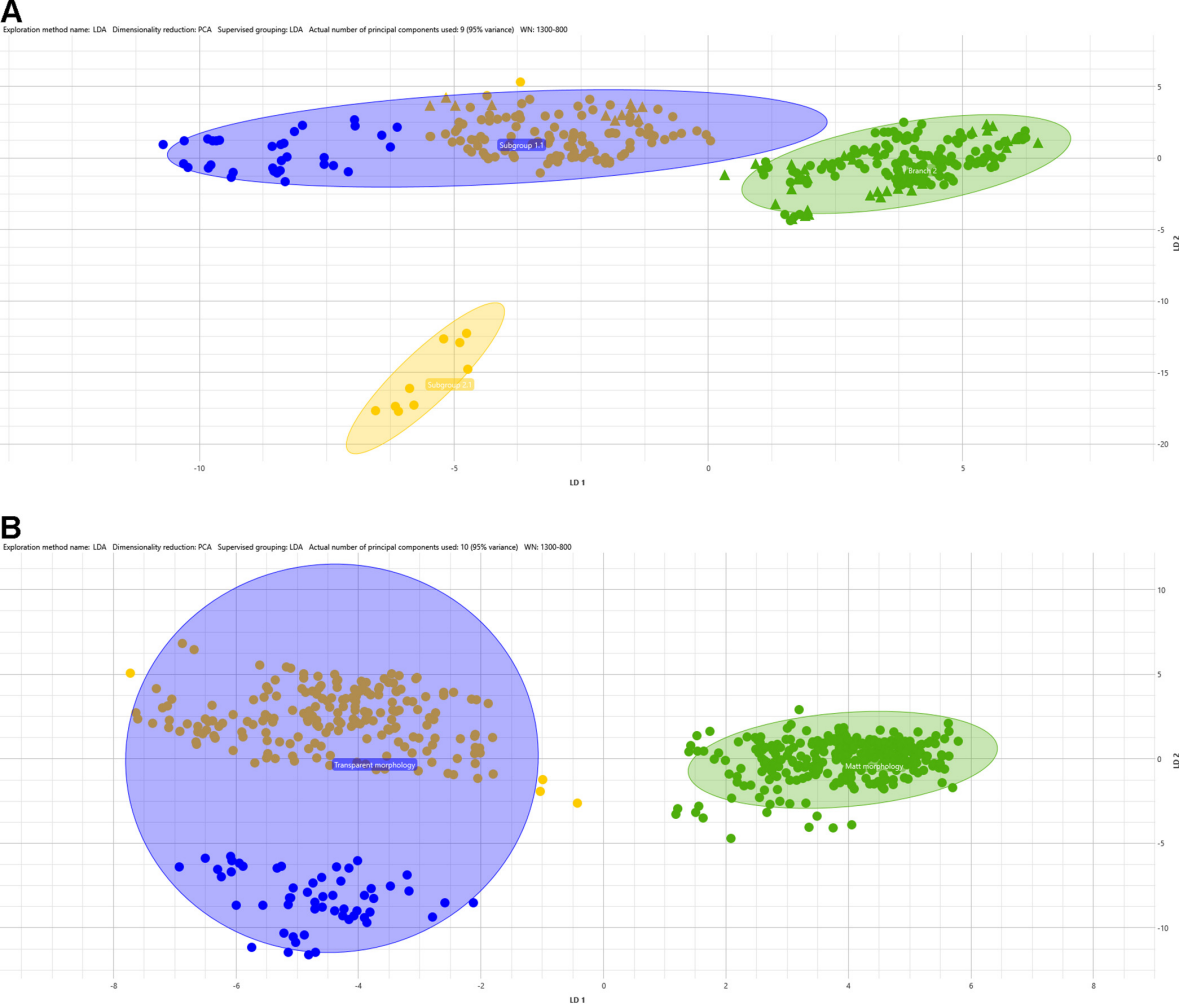
LDA supervised analysis. Fifty-three *M. chimaera* isolates were grouped by hospital source (A) and by colony morphology (B). Hospital origin is indicated by color: yellow for hospital A; green for hospital M; and blue for hospital S.

Environmental isolates belonging to the three different hospitals generated three different, distinct clusters. Figure 1A shows *M. chimaera* isolates from hospital A in yellow, hospital S in blue, and hospital M in green. The sum of the variance of the first two principal components in linear discriminant analysis (LDA) covers 79.17%.

*M. chimaera* isolates exhibited two kinds of colony morphology on 7H11 solid media: transparent and matte. All isolates defined as “transparent” had translucent, shiny colonies, while “matte” isolates had larger opaque colonies. Both types of morphology were acid-alcohol-fast bacilli using Ziehl-Neelsen stain, without significant differences in microscopic observation. LDA analysis grouped by *M. chimaera* colony morphology produced two separated clusters: isolates from hospital A and S, which exhibited transparent morphology, are grouped together in the first, while *M. chimaera* matte colonies from hospital M are clustered in the second (Fig. 1B).

### WGS results.

The WGS analysis was carried out on 33 of the 53 *M. chimaera* isolates, while the remaining 20 strains were not analyzed as they were collected from the same positive HCU but from different circuits (*n* = 11), or collected within a few days of each other from the same positive HCU (*n* = 9), as shown in Table 2.

**TABLE 2:** Results of IR-Biotyper and WGS analysis

Hospital	HCU	Date	IR-Biotyper CLUSTER by hospital source	Sequence ID	WGS classification
A	Maquet 1	20/11/2018	Cluster 1	SP1274	Ungrouped
	Maquet 2	20/11/2018	Cluster 1	SP1261	1.1
	Stockert 1	28/11/2018	Cluster 1	SP1263	1.1
		01/02/2019	Cluster 1	SP1563	1.1
	Stockert 2	20/11/2018	Cluster 1	SP1260	1.1
		28/11/2018	Cluster 1		
	Stockert 3	22/11/2018	Cluster 1	SP1257	1.1
		28/11/2018	Cluster 1		
	Stockert 6	23/11/2018	Cluster 1	SP1259	1.1
		28/11/2018	Cluster 1		
		30/11/2018	Cluster 1		
		01/02/2019	Cluster 1	SP1567	1.1
		27/02/2019	Cluster 1		
		Date of returning machine after deep disinfection: 03/10/2019			
		23/01/2020	Cluster 1	SP1395	2.1
	Stockert 7	28/11/2018^*a*^	Cluster 1	SP1248	1.1
		28/11/2018^*a*^	Cluster 1		
		30/11/2018	Cluster 1		
		01/02/2019	Cluster 1	SP1562	1.1
		27/02/2019	Cluster 1		
		Date of returning machine after deep disinfection: 03/10/2019			
		17/12/2019	Cluster 1	SP1394	1.1
	Stockert 9	11/03/2020	Cluster 1	SP1405	1.1
M	Stockert 4	02/03/2020^*a*^	Cluster 2	SP1398	Branch 2
		02/03/2020^*a*^	Cluster 2		
	Stockert 5	06/04/2020^*a*^	Cluster 2	SP1402	Branch 2
		06/04/2020^*a*^	Cluster 2		
	Stockert 8	02/03/2020	Cluster 2	SP1401	Branch 2
	Stockert 11	06/07/2020	Cluster 2	SP1475	Ungrouped
	Stockert 12	02/03/2020	Cluster 2	SP1400	Branch 2
	Stockert 13	18/09/2019	Cluster 2	SP1254	Branch 2
	Stockert 14	04/05/2020^*a*^	Cluster 2	SP1403	Branch 2
		04/05/2020^*a*^	Cluster 2		
	Stockert 15	08/06/2020	Cluster 2	SP1473	Ungrouped
	Stockert 16	04/05/2020^*a*^	Cluster 2	SP1404	Branch 2
		04/05/2020^*a*^	Cluster 2		
	Stockert 17	03/02/2020^*a*^	Cluster 2	SP1396	Branch 2
		03/02/2020^*a*^	Cluster 2		
	Stockert 18	06/11/2019	Cluster 2	SP1256	Branch 2
	Stockert 19	05/10/2020^*a*^	Cluster 2	SP1478	Ungrouped
		05/10/2020^*a*^	Cluster 2		
	Stockert 20	18/09/2019	Cluster 2	SP1414	Branch 2
		07/10/2019	Cluster 2		
	Stockert 21	18/09/2019^*a*^	Cluster 2	SP1253	Branch 2
		18/09/2019^*a*^	Cluster 2		
		07/10/2019	Cluster 2		
	Stockert 22	07/10/2019^*a*^	Cluster 2	SP1255	Branch 2
		07/10/2019^*a*^	Cluster 2		
	Stockert 24	02/03/2020	Cluster 2	SP1399	Branch 2
	Tap water	03/05/2021	Cluster 2	SP1565	Branch 2
S	Stockert 10	18/09/2019^*a*^	Cluster 3	SP1570	1.1
		18/09/2019^*a*^	Cluster 3		
		07/10/2019^*a*^	Cluster 3	SP1571	1.1
		07/10/2019^*a*^	Cluster 3		
	Stockert 23	03/02/2020	Cluster 3	SP1418	1.1

a Sample was collected from different circuit (patient or cardioplegia).

The WGS analysis on *M. chimaera* isolates allowed all 33 strains to be subtyped into groups and subgroups, using the classification proposed by van Ingen and collaborators (17). Thirteen strains were from samples collected at hospital A, three at hospital S, and 17 at hospital M (including one from prefilter tap water).

Strain results were then associated with the three hospital sites where the original water samples were collected. At hospital A, among the 13 typed strains, 11 were classified as belonging to subgroup 1.1 (with single-nucleotide polymorphisms [SNPs] 209278 G > A and 113518 G > A); one strain did not show any classifying mutations and was reported as ungrouped, and one strain was classified as subgroup 2.1 (3022332 T > C, 3406341 C > T, 1828053 C > T).

Fourteen of the 17 typed strains, isolated from samples collected at hospital M were classified as Branch 2 (SNPs 5003561 A > G, 2339764 C > T), and the remaining three were reported as ungrouped. No 1.1 strains were found in samples from this hospital site.

All of the three typed strains, isolated from samples collected at hospital S, belonged to the subgroup 1.1 (with SNPs 209278 G > A and 113518 G > A).

**FIG 2:**
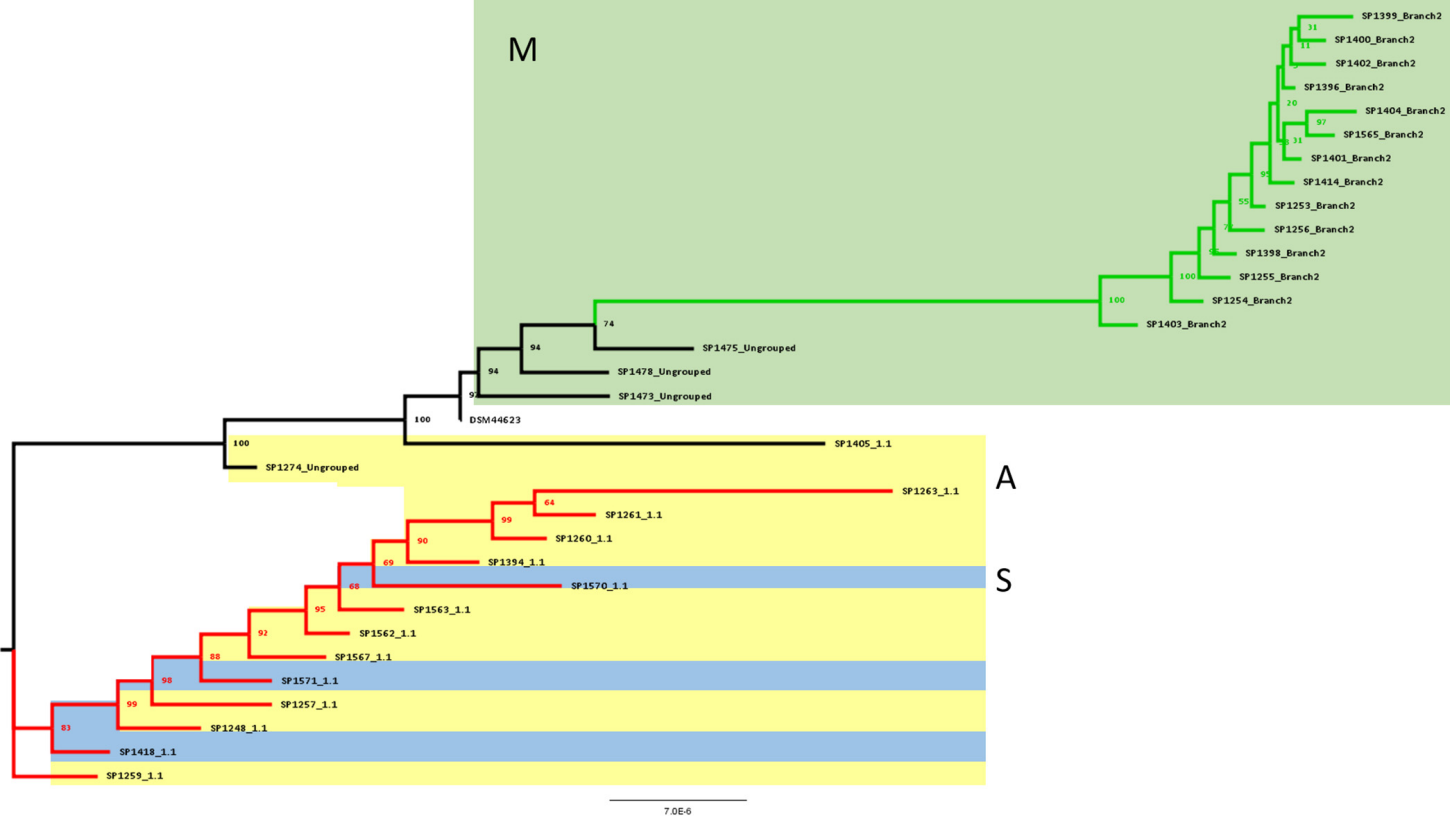
ML tree on WGS (~5.8 Mb). WGS analysis of 33 *M. chimaera* isolates from hospitals M (green area), A (yellow area), and S (blue area). Branches with different colors highlighted the 1.1 and branch 2 groups (red for subgroup 1.1 and green for branch 2). The strain typed as subgroup 2.1 is not shown for graphical reasons.

In the maximum-likelihood tree generated (Fig. 2), the strains found at hospital M, belonging to branch 2, were significantly enclosed in only one clade (100% bootstrap). In addition, the 1.1 strains from hospitals A and S showed a strong similarity, sharing a significant node (>90% bootstrap).

### IR Biotyper versus WGS analysis.

**FIG 3:**
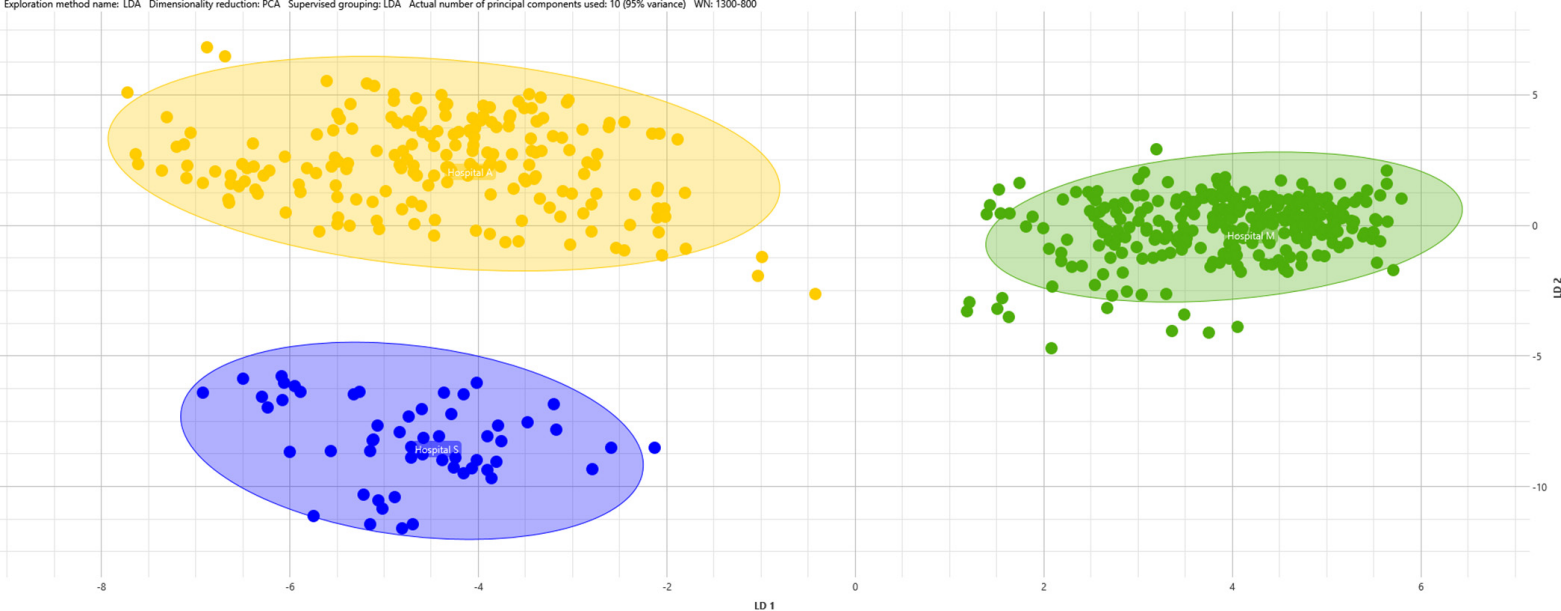
LDA supervised analysis of 33 *M. chimaera* isolates grouped by WGS results. Circles represent defined WGS strains (subgroup 1.1, 2.1, and branch 2), while triangles represent ungrouped strains. Colored area represent IR Biotyper clustering while hospital origin is shown by the color of the circles or triangles: yellow for hospital A; green for hospital M; and blue for hospital S.

Figure 3 shows the LDA of three well-separated clusters, which is consistent with the results of WGS, where it is possible to distinguish subgroup 1.1 (blue area), subgroup branch 2 (green area), and subgroup 2.1 (yellow area), while the ungrouped strains (depicted as triangles) are spread throughout the graph. The sum of the variance of the first two principal components in LDA covers 80.45%.

A summary of IR Biotyper results and WGS analysis *of M. chimaera* isolates used in this study are shown in Table 2, by hospital, HCU (or tap water), and date of sampling.

## DISCUSSION

This is the first study to evaluate the efficacy of IR-Biotyper system to identify clusters of M. chimaera, and to compare the results with WGS analysis, the reference method currently used to determine whether a clinical strain is related to an HCU outbreak strain (23, 24).

The study was carried out on a large number of HCU water samples (*n* = 440) and hospital tap water samples (*n* = 23) used to fill the HCU tanks, longitudinally collected during the period 2018 to 2021 from three Italian hospitals and investigated for the presence of *M. chimaera* according to ECDC guidelines (10). Overall, 50% of the HCUs sampled were contaminated by *M. chimaera*, in particular 18.2% (2/11) of the HCU40 and 58.5% (24/41) of the Stockert 3T models. This results seem to indicate that the Stockert 3T is more easily contaminated than the HCU40 model. In our opinion, this could be due to both the different HCU design and the different disinfection protocols used.

Device design is thoroughly investigated by Widmer and collaborators: the position of the ventilators and water tanks, as well as the direction of airflow, differ substantially between the two types of HCU. Furthermore, in the HCU40 model the temperature of the water tank was kept continuously at 2°C to 4°C, while water remains at ambient temperature in the 3T HCU, potentially allowing *M. chimaera* to grow faster and/or to a higher density (25).

The disinfection procedures of each model were supplied by the manufacturers, and the protocols were carefully followed at each hospital, noting the operator’s name, date, and reagents used for disinfection. The procedures for 3T HCU included disinfection with peracetic acid (Puristeril 340, Fresenius Medical Care, Bad Homburg, Germany) every 2 weeks and weekly water exchange with the addition of 100 mL 3% hydrogen peroxide (26).

Disinfection of the HCU40 was performed with 2% tosylchloramide sodium, known as Chloramine-T, once a week and water was exchanged every 2 weeks.

Decontamination procedures could have different effects on *M. chimaera* biofilm removal. Most sanitizing treatments are corrosive and encourage the formation of crevices which facilitate bacterial nesting and growth. Furthermore, the elimination of viable bacteria does not necessarily lead to the removal of biofilm matrix, as shown by Totè and collaborators (13, 27).

A large number of HCUs (*n* = 52) were monitored because HCUs that are colonized by *M. chimaera* need to be sent back to the manufacturer, then deeply sanitized, and returned with a Mycobacteria-free certificate. In the meantime, colonized HCUs were temporarily replaced with a certified Mycobacteria-free device from the manufacturer. These devices are equally likely to arrive in each hospital.

All tap water samples collected after the absolute filter (*n* = 20) were negative. One sample of prefilter tap water was collected at each hospital site: the plumbing system was positive for *M. chimaera* in hospital M, while hospitals A and S tested positive for M. gordonae. Fifty-two HCU water samples were positive for *M. chimaera*, two for M. chelonae, and one for *M. xenopy*.

M. gordonae and *M. paragordonae* were found irregularly throughout the HCUs (not reported). Therefore, mixed Mycobacteria MGIT were subcultured in 7H11 solid media in order to obtain pure *M. chimaera* colonies for reliable sequencing and IR-Biotyper results.

The IR Biotyper system was used for clustering of *M. chimaera* isolated from hospitals located in three Italian provinces of the Emilia-Romagna Region. Using a statistical approach of dimensionality reduction by LDA, we obtained three separate clusters of *M. chimaera*, ascribable to each hospital. This nonrandom distribution leads to the hypothesis that *M. chimaera* subtypes circulating in the local plumbing can colonize HCUs, in contrast with the current hypothesis that contamination occurs in the HCU production site (17, 24). Our findings are supported by the fact that two contaminated HCUs, Stockert 6 and Stockert 7, were positive for *M. chimaera* shortly (75 and 112 days, respectively) after returning from deep disinfection at the manufacturer’s site. The strains found after cleaning showed a similar genetic profile and the same Biotyper cluster of those isolated prior to disinfection.

Interestingly, in this study we observed two *M. chimaera* colony morphotypes: all isolates from hospital A and hospital S had a transparent morphology, while isolates from hospital M were opaque. This result is in line with the hypothesis that different *M. chimaera* strains can circulate in the hospital water distribution system.

According to the genome-based phylogenies (WGS) and following the classification proposed by van Ingen and collaborators (17), three distinct *M. chimaera* groups appear to have contaminated the HCU water systems: subgroups 1.1 (*n* = 14), 2.1 (*n* = 1), and Branch 2 (*n* = 14). Furthermore, four strains did not show any classifying mutation and were reported as ungrouped. Based on the WGS results, we showed that the *M. chimaera* strains isolated from HCUs at hospital M, almost all belonging to branch 2, were significantly enclosed in only one clade. Moreover, most of the strains isolated from HCUs at hospitals A and S share a highly similar genetic profile and were classified into subgroup 1.1, which was originally associated with the cardiac surgery outbreak. Although we cannot draw any definitive conclusion, we can envision a common source for the branch 2 strains isolated at hospital M. Similarly, subgroup 1.1 strains isolated at both hospitals A and S could share the same origin. Furthermore, the only *M. chimaera-*positive sample from hospital M prefilter tap water was clustered with the isolates from the HCU of the same hospital (branch 2), confirming that the plumbing system could also represent the source of HCU contamination and, potentially, patient infection. Literature data report the almost exclusive presence of subtypes belonging to group 1 (subtypes 1.1 and 1.8) in patients with *M. chimaera* HCU-related infection. We can, therefore, speculate that these subtypes are more virulent, while HCUs contaminated with branch 2 subtypes expose the patients to a lower risk (17, 24).

Limits of this study were the small number (*n* = 3) of prefilter hospital tap water collected in the surgery area and the small amount (1 L) of postfilter water samples which did not permit detection of *M. chimaera* at each hospital site. Increasing the amount of postfilter tap water collected (e.g., 8 L), could permit to improve sensitivity.

Future studies are necessary to understand how *M. chimaera* found in the hospital water system is able to pass through the absolute filter to contaminate the HCUs or other medical equipment such as endoscope reprocessing devices or hemodialysis systems.

In conclusion, although WGS is considered the standard method for the analysis of *M. chimaera* strains isolated from patients and HCU for epidemiological investigations, the results obtained in this preliminary study with IR-Biotyper system indicate that integration of these two techniques could represent a model for pathogens typing during environmental assessment, clinical surveillance, or outbreak events.

## MATERIALS AND METHODS

### Samples collection.

This study collected a 1-L HCU water sample at monthly intervals between October 2018 and May 2021 from three Italian hospitals (hospital A, hospital S, and hospital M). Samples were collected from 52 HCUs: 41 devices were Stockert 3T model (Livanova, Germany) and 11 were HCU40 model (Maquet, Germany). A total of 417 water samples were collected from HCUs over the study period. Furthermore, hospital tap water, collected in the surgery area and used to fill the HCUs tanks, was also tested. A total of 21-L samples were collected after the absolute filter (0.22 μm pore size), whereas one prefilter sample was taken in each hospital (this time 8 L) to improve sensitivity.

### Sample processing.

All samples were processed at the Microbiology Unit, IRCCS Azienda Ospedaliero-Universitaria of Bologna, Italy. Samples were concentrated by cellulose nitrate membrane (0.45 μm), using Microsart filtration system (Sartorius, Germany) and resuspended in 10 mL of 0.9% saline solution (final dilution 1:100).

Concentrated samples were digested and decontaminated, using BBL MycoPrep solution (Becton Dickinson, USA) according to the manufacturer’s instructions, and resuspended in 2 mL of phosphate buffered solution (28). Then, decontaminated samples were inoculated onto solid medium (Lowenstein-Jensen, Heipha Diagnostics, Germany), and into Middlebrook 7H9 Broth (MGIT, Becton Dickinson, USA), according to the routine workflow for Mycobacteria detection. MGIT incubation was performed on MGIT 960, a fully automated system for the rapid detection of Mycobacteria, while Lowenstein-Jensen were incubated at 37°C, without CO2. Solid and liquid cultures were considered negative after 42 days of incubation without isolation of any Mycobacteria, while positive cultures were verified for acid-fast bacilli by microscopic examination using Ziehl-Neelsen stain. The possible isolation of other bacteria has been obtained from other laboratories of which we are not aware of the results.

### Isolate collection.

Mycobacteria from positive MGIT cultures were first identified as NTM by Genotype CM (Bruker, Germany) and then identified as *M. chimaera* by Genotype NTM-DR (Bruker, Germany). Positive MGIT were subcultured in Middlebrook 7H11 Agar (7H11 plate, Becton Dickinson, USA) for approximately 2 weeks, in order to obtain cultures of “pure” *M. chimaera*. All the isolated *M. chimaera* strains were stored at −20°C using Microbank cryovial (Pro-Lab Diagnostics, Canada), after further identification by matrix-assisted laser desorption ionization-time-of-flight mass spectrometry using MALDI Biotyper system (Bruker, Germany).

As requested by the Italian Ministry of Health as part of the national surveillance program, unfrozen *M. chimaera* strains were subcultured in a new MGIT and sent to the National Institute from Infectious Diseases “L. Spallanzani” (INMI) to carry out molecular epidemiological investigation. Subsequent DNA extraction, WGS, and bioinformatic analysis were then performed at INMI.

### Sample preparation for FTIR Biotyper analysis.

Frozen *M. chimaera* strains were thawed and subcultured on 7H11 plates for 3 weeks. Colony morphology was also recorded. The FTIRS was performed by IR Biotyper platform (Bruker, Germany) and samples were prepared according to the manufacturer’s instructions.

Briefly, three overloop inocula of each strain were taken directly from the confluent part of the 7H11 plate using a 1-μL disposable inoculation loop and resuspended in the suspension vial with 50 μL of 70% (vol/vol) ethanol. Then, 50 μL of deionized water were added to each vial and 15 μL of homogenized suspension were pipetted onto four spots (technical replicates) of the silicon sample plate. The sample plate was incubated at 37°C for 15 min and then read by IR Biotyper platform.

### Spectra acquisition and analysis.

Spectra were acquired and processed by the OPUS software V7.5.18 and the IR Biotyper Client Software V3.0 (Bruker, Germany), with the default settings recommended by the manufacturer. The laser emits red light with a wavelength of 633 nm, and the acquisition spectral range was between 500 and 4,000 cm^−1^. The second derivative was calculated, and data corresponding to the polysaccharide’s absorption region (800 to 1,300 cm-^1^) were vector normalized. Similarity analysis (or dimensionality reduction) was determined by principal component analysis (PCA), and grouping was supervised by LDA. LDA was performed using at least 10 principal components (PCs) to achieve 95% variance. To perform the LDA, three biological replicates were analyzed for each isolate from fresh culture (3 weeks long), starting each time from the frozen sample. Two IR test standards (IRTS1 and 234 IRTS2), spotted in duplicate, were also included as controls.

### Sample preparation for WGS.

Genomic DNA of *M. chimaera* strains was extracted from MGIT subcultures according to the manufacturer’s protocol using QIAamp DNA minikit (Qiagen, Hilden, Germany). Before sequencing, DNA was quantified by Qubit 4.0 using the Qubit dsDNA HS assay kit (Thermo Fisher Scientific, USA) and subsequently processed, as described in the manufacturer’s protocol for WGS using the Ion Xpress Plus Fragment Library Kit (Thermo Fisher Scientific, USA) for sample library preparation. The Chef and S5 platforms were used for automated chip preparation and sequencing, respectively, producing 250-bp reads.

### WGS analysis.

Genomic DNA, extracted from *M. chimaera* strains, was sequenced using the Ion Torrent sequencing system, as described above. The readings’ quality was evaluated using FastQC software and only the fastq files with high-quality standard were analyzed. The readings included in the fastq files were mapped to the reference genome DSM-44623 sequence (NZ CP015278.1) (1), using BWA v0.7.17-r1188 (29) and samtools v1.9 (30). In particular, bwa mem was used to perform an alignment processing, while samtools fixmate, sort, and index functions were used to provide bam files for the variant calling step.

Variant calling was performed with freebayes v1.3.2 (Erik Garrison, Gabor Marth Haplotype-based variant detection from short-read sequencing. arXiv:1207.3907; https://arxiv.org/abs/1207.3907).

The SNP variants were filtered, imposing the following threshold variant parameters: haploid genome, only SNPs, 5-fold minimum coverage, 90% allele frequency, 50 mapping quality, and 30 base quality (-F 0.9 -p 1 -i -X -u -m 50 -q 30 –min-coverage 5).

The phylogenetic relationships between genomic sequences were inferred by IQTREE version 2.0, based only on SNP variability along the whole genome (~5.8 Mb) (31). The maximum-likelihood tree was built from multisequence alignment, assessing the significance of the nodes by bootstrap analysis with 10,000 replicates (>80% considered significant). The best mutation model was selected through the Model Finder function of IQTREE, selecting HKY+F+I model (Hasegawa, Kishino and Yano model, based on unequal transition rates and unequal base frequencies, basing on empirical frequencies and allowing for a proportion of invariable sites) (32). The tree was plotted and adapted by Figtree v1.4.3 (33).

The group and subgroup classification of strains was carried out according to the method described by van Ingen and collaborators (17).

### Data availability.

All raw data generated were submitted to the Sequence Read Archive (SRA) under the BioProject accession number PRJNA554584.
